# Based on Cardiopulmonary Exercise Testing to Construct and Validate Nomogram of Long‐Term Prognosis Within 12 Months for NSCLC

**DOI:** 10.1111/crj.13806

**Published:** 2024-08-08

**Authors:** Xinyu Wang, Jin Li, Jingjie Zhou, Min Gao, Bin Wang, Yiman Tong, Yuhan Cao, Wei Chen

**Affiliations:** ^1^ Department of Rehabilitation Medicine Xuzhou Rehabilitation Hospital Affiliated to Xuzhou Medical University Xuzhou China; ^2^ Department of Rehabilitation Medicine, Xuzhou Central Hospital The Xuzhou Clinical College of Xuzhou Medical University Xuzhou China

**Keywords:** cardiopulmonary exercise testing, clinical decision, nomogram, non‐small cell lung cancer, prognosis

## Abstract

**Objective:**

Construction nomogram was to effectively predict long‐term prognosis in patients with non‐small cell lung cancer (NSCLC).

**Materials and Methods:**

The nomogram is developed by a retrospective study of 347 patients with NSCLC who underwent cardiopulmonary exercise testing (CPET) before surgery from May 2019 to February 2022. Cross‐validation divided the data into a training cohort and validation cohort. The discrimination and accuracy ability of the nomogram were proofed by concordance index (C‐index), calibration curve, receiver operating characteristic (ROC) curve, the area under the curve (AUC), and time‐dependent ROC in validation cohort.

**Results:**

Age, intraoperative blood loss, VO_2_ peak, and VE/VCO_2_ slope were included in the model of nomogram. The model demonstrated good discrimination and accuracy with C‐index of 0.770 (95% CI: 0.712–0.822). AUC of 6 (AUC: 0.789, 95% CI: 0.726–0.851) and 12 months (AUC: 0.787, 95% CI: 0.724–0.850) were shown in ROC. Time‐independent ROC maintains a good effect within 12 months.

**Conclusion:**

We developed a nomogram based on CPET. This model has a good ability of discrimination and accuracy. It could help clinicians to make treatment decision in clinical decision.

## Graphical abstract

Currently none developed nomogram considers cardiopulmonary exercise testing parameter. We first time construct a nomogram included age, intraoperative blood loss, VO_2_ peak, and VE/VCO_2_ slop. The model demonstrated good discrimination and accuracy with C‐index of 0.770 (95% CI: 0.712–0.822). AUC of 6 (AUC: 0.789, 95% CI: 0.726–0.851) and 12 months (AUC: 0.787, 95% CI: 0.724–0.850) were shown in ROC. Time‐independent ROC maintain a good effect within 12 months. This model with good ability of discrimination and accuracy could help clinicians make treatment decision.

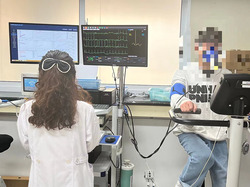

## 1 Introduction

According to Cancer Statistics 2023, lung cancer is the primary mortality cancer, and it is 2.5 times higher than the second leading cause of death (colorectal cancer), in which non‐small cell lung cancer (NSCLC) accounts for 85% [1, 2]. The choice of treatment for lung cancer includes surgery, radiotherapy, and chemotherapy; however, surgery is the best option when available especially for NSCLC [3]. Video‐assisted thoracic surgery (VATS) has been widely used in clinical practice and could get better prognosis than open thoracotomy [4]. However, various postoperative complications still remain high at 15.8%–31.7% [5]. Postoperative complications have a negative effect on mortality [6]. Hence, how to reduce the risk of postoperative complications is an important problem of public health.

Cardiopulmonary exercise testing (CPET) is the “gold standard” for evaluating cardiopulmonary function. According to the American College of Chest Physicians, the European Association for Cardiovascular Prevention & Rehabilitation, and the American Heart Association guidelines [7, 8], CPET can be considered as the preoperative assessment to assess the risk of surgery to guarantee postoperative prognosis. Nomogram could be considered as a statistical tool that can visualize the results of multifactor regression analysis and make the results readable [9]. At present, the model of nomogram has been based on the clinical data of NSCLC to establish [2, 10, 11], but in our knowledge, no nomogram considered the factor of CPET. Thus, our object was to construct nomogram of NSCLC based on CPET to predict long‐term prognosis.

## 2 Method

### 2.1 Study Design and Population

This is a retrospective clinical study. NSCLC patients who received CPET before hospitalization in the Department of Thoracic Surgery of Xuzhou Central Hospital from May 2019 to February 2022 were included. The inclusion criteria are as follows: (1) postoperative pathological confirmation of the NSCLC, (2) age > 30 years old, and (3) communicating normally and consciously normal. The exclusion criteria are as follows: (1) lower limbs could not pedal cycle ergometer due to limited movement, (2) participated in other lung cancer‐related research projects before examination recently, and (3) patients with related contraindications were excluded [12]. The study was approved by Biomedical Research Ethics Review Committee of Xuzhou Central Hospital (XZXY‐LK‐20221201‐113). Because this is a retrospective study, informed consent was waived.

Baseline clinical data including age, body mass index (BMI), sex, exercise habits, smoking, histology, radiochemotherapy, comorbidity, pathological stage, and intraoperative blood loss were recorded. According to previous studies [13, 14] and the International Classification of Diseases, 10th revision (ICD‐10) (https://icd.who.int/browse10/2016/en), we recorded postoperative cardiopulmonary complications (PCCs) including pulmonary edema, atelectasis, emphysema, hemothorax, empyema, chylothorax, bronchopleural fistula, respiratory failure, postoperative pneumonia (imaging findings of increased plaque or flake high‐density image), adult respiratory distress syndrome, pulmonary torsion, esophagopleural fistula, pulmonary embolism, arrhythmia, and acute coronary syndrome within 12 months from our hospital database. In addition, we call patients every 3 months for follow‐up in the period of 12 months.

### 2.2 Pulmonary Function Test (PFT) and CPET

PFT and CPET recorded the patients’ cardiopulmonary function. Forced expiratory volume in 1 s (FEV1), FEV1/predicted (FEV1%), forced vital capacity (FVC), FVC%, and FEV1/FVC were recorded from PFT. We used ramp protocol to gradually increase work rate (WR) (10–20 W/min) until exhaustion. Patients were monitored by 12‐lead electrocardiogram, oximeter, and sphygmomanometer during exercise. We collected minute ventilation at peak (VE peak), VE peak%, WR peak, WR peak%, VO_2_ at anaerobic threshold (VO_2_ AT), VO_2_ peak, VO_2_ peak%, kilogram oxygen uptake at (VO_2_/kg AT), VO_2_/kg peak, carbon dioxide output at peak (VCO_2_ peak), systolic blood pressure at peak (SBP peak), diastolic blood pressure at peak (DBP peak), O_2_ pulse peak, O_2_ pulse peak%, the minute ventilation and oxygen production (VE/VO_2_ peak), and the minute ventilation to carbon dioxide production slope (VE/VCO_2_ slope).

### 2.3 Nomogram Construction and Statistical Analyses

The factor *p* < 0.2 was included in the Cox regression analysis by univariate analysis. The multivariate Cox regression was applied to calculate hazard ratio (HR) and 95% confidence interval (95% CI) to ensure influential variables for PCCs. SPSS 23.0 was used for statistical analysis. Then, we used these variables to construct nomogram for predicting PCCs through R software Version 4.1.2. Cross‐validation divided the data into a training cohort and validation cohort. The model of accuracy and discrimination were measured by concordance index (C‐index), calibration curve, receiver operating characteristic (ROC) curve, the area under the curve (AUC), and time‐dependent ROC in validation cohort. Two‐sided *p* < 0.05 was considered statistically significant.

## 3 Result

### 3.1 Clinical Data

A total of 341 patients with NSCLC were included. Among them, 200 (58.7%) patients were ≥ 60 years and 169 (49.6%) were male. Stage I was performed in 285 (83.6%), Stage II in 36 (10.6%), and Stage III in 20 (5.8%). Two (0.7%) patients underwent pneumonectomy, 209 (61.3%) patients underwent lobectomy, and 130 (38.1%) patients underwent segmentectomy. The relevant data of patients are shown in Table 1.

**TABLE 1:** Demographic and clinical characteristics of patients with NSCLC.

Variable	All (*n* = 341)	With (*n* = 65)	Without (*n* = 276)	*t/z/χ* ^2^	*p* value
Age (*n*, %)					
≥ 60	200 (58.7%)	55 (84.6%)	145 (52.5%)	22.324	< 0.001
< 60	141 (41.3%)	10 (15.4%)	131 (47.5%)		
BMI	24.22 ± 3.03	24.30 ± 3.12	24.20 ± 3.01	0.239	0.811
Sex (*n*, %)					
Male	169 (49.6%)	35 (53.8%)	134 (48.6%)	0.186	0.666
Female	172 (50.4%)	30 (46.2%)	142 (51.4%)		
Smoking (*n*, %)					
No	196 (57.5%)	34 (52.4%)	162 (58.7%)	0.978	0.613
Yes	106 (31.1%)	22 (33.8%)	84 (30.4%)		
Former	39 (11.4%)	9 (13.8%)	30 (10.9%)		
Exercise habit (*n*, %)					
Yes	195 (57.2%)	35 (53.8%)	160 (58%)	0.366	0.545
No	146 (42.8%)	30 (46.2%)	116 (42%)		
Stage (*n*, %)					
I	285 (83.6%)	49 (75.4%)	236 (85.5%)	4.299	0.108
II	36 (10.6%)	11 (16.9%)	25 (9.1%)		
III	20 (5.8%)	5 (7.7%)	15 (5.4%)		
Histology (*n*, %)					
AdC	299 (87.7%)	49 (75.4%)	250 (90.6%)	10.531	0.003
SqCC	33 (9.7%)	13 (16.9%)	20 (7.2%)		
Other	9 (2.6%)	3 (7.7%)	6 (2.2%)		
Type of surgery (*n*, %)					
Pneumonectomy	2 (0.6%)	0 (0%)	2 (0.7%)	6.731	0.028
Lobectomy	209 (61.3%)	49 (75.4%)	160 (58%)		
Segmentectomy	130 (38.1%)	16 (24.6%)	114 (41.3%)		
Radiochemotherapy (*n*, %)					
Yes	97 (28.4%)	27 (41.5%)	70 (25.4%)	6.763	0.009
No	244 (71.6%)	38 (58.5%)	206 (74.6%)		
CAD (*n*, %)					
Yes	49 (14.4%)	14 (21.5%)	35 (12.7%)	3.354	0.067
No	292 (85.6%)	51 (78.5%)	241 (87.3%)		
COPD (*n*, %)					
Yes	72 (21.1%)	20 (30.8%)	52 (18.8%)	4.494	0.034
No	269 (78.9%)	45 (69.2%)	224 (81.2%)		
DM (*n*, %)					
Yes	38 (11.1%)	9 (13.8%)	29 (10.5%)	0.592	0.442
No	303 (88.9%)	56 (86.2%)	247 (89.5%)		
Intraoperative blood loss (mL)	100 (50)	100 (50)	100 (100)	4.994	< 0.001

Abbreviations: AdC = adenocarcinoma; BMI = body mass index; CAD = coronary artery disease; COPD = chronic obstructive pulmonary disease; DM = diabetes mellitus; SqCC = squamous cell carcinoma.

### 3.2 PCCs

**FIGURE 1:**
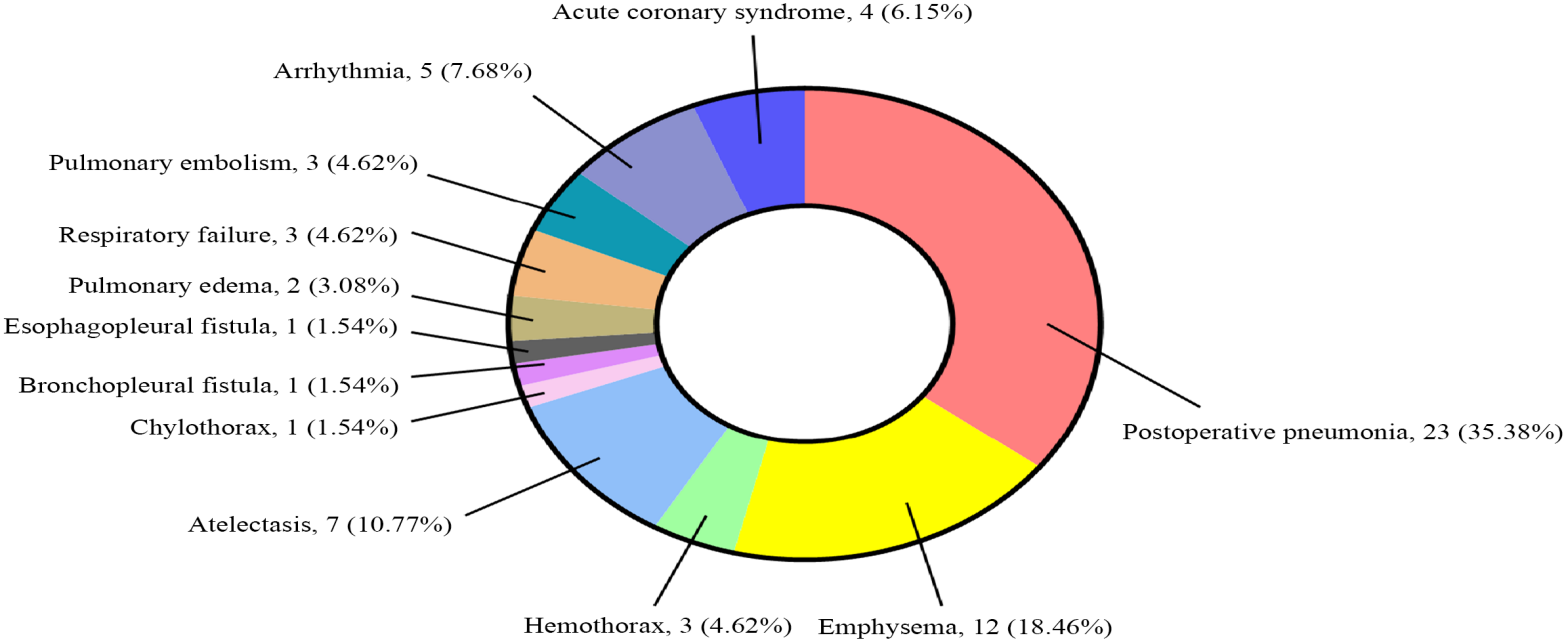
The result of PCCs for 65 patients.

Sixty‐five patients have PCCs: 23 (35.38%) in postoperative pneumonia, 12 (18.46%) in emphysema, 3 (4.62%) in hemothorax, 7 (10.77%) in atelectasis, 1 (1.54%) in chylothorax, 1 (1.54%) in bronchopleural fistula, 1 (1.54%) in esophagopleural fistula, 2 (3.08%) in pulmonary edema, 3 (4.62%) in respiratory failure, 3 (4.62%) in pulmonary embolism, 5 (7.68%) in arrhythmia, and 4 (6.15%) in acute coronary syndrome. The results of PCCs are given in Figure 1.

### 3.3 PFT and CPET

As shown in Table 2, FEV1, FVC, WR AT, WR peak, VO_2_ AT, VO_2_ peak, VO_2_ peak%, VO_2_/kg AT, VO_2_/kg peak, VCO_2_, O_2_/HR peak, O_2_/HR peak%, VE/VO_2_ peak, and VE/VCO_2_ slope were statically different.

**TABLE 2:** Characteristics of patients with NSCLC in PFT and CPET.

Variable	With (*n* = 65)	Without (*n* = 276)	*T*	*p* value
FEV1	2.21 ± 0.61	2.46 ± 0.71	2.689	0.008
FEV1%	97.48 ± 19.83	99.98 ± 18.47	0.970	0.333
FVC	2.64 ± 0.65	2.85 ± 0.75	2.139	0.033
FVC%	92.65 ± 15.86	93.88 ± 14.75	0.596	0.551
FEV1/FVC	83.98 ± 11.19	86.21 ± 9.33	1.670	0.096
VE peak	40.78 ± 10.30	43.18 ± 11.39	1.555	0.121
VE peak%	59.62 ± 13.94	58.82 ± 12.36	0.458	0.647
WR AT	63.23 ± 18.10	75.32 ± 22.29	4.064	< 0.001
WR peak	94.00 ± 24.52	110.03 ± 31.12	4.487	< 0.001
WR peak%	100.80 ± 21.76	105.66 ± 19.83	1.746	0.082
VO_2_ AT	762.18 ± 184.37	880.13 ± 242.78	3.674	< 0.001
VO_2_ peak	1061.26 ± 242.42	1238.09 ± 328.50	4.914	< 0.001
VO_2_ peak%	71.92 ± 13.73	77.35 ± 13.92	2.833	0.005
VO_2_/kg AT	11.84 ± 2.53	13.60 ± 3.14	4.191	< 0.001
VO_2_/kg peak	16.40 ± 3.34	19.01 ± 3.79	5.097	< 0.001
VCO_2_ peak	1217.48 ± 320.00	1433.61 ± 419.97	4.593	< 0.001
O_2_/HR peak	8.36 ± 1.87	9.13 ± 2.45	2.372	0.018
O_2_/HR peak%	86.34 ± 14.87	91.14 ± 16.92	2.104	0.036
SBP peak	185.18 ± 26.07	187.18 ± 26.74	0.543	0.587
DBP peak	81.60 ± 13.87	85.02 ± 14.68	1.707	0.089
VE/VO_2_ peak	35.91 ± 4.54	33.22 ± 4.75	4.146	< 0.001
VE/VCO_2_ slope	30.62 ± 4.52	26.91 ± 3.46	6.199	< 0.001

Abbreviations: AT = anaerobic threshold; DBP = diastolic blood pressure; FEV1 = forced expiratory volume in 1 s; FVC = forced vital capacity; SBP = systolic blood pressure; VCO_2_ = carbon dioxide output; VE = minute ventilation; VO_2_ = oxygen uptake; VO_2_/HR = oxygen pulse; WR = work rate.

### 3.4 Multivariate Cox Regression

The Cox regression shows that age, intraoperative blood loss, VO_2_ peak, and VE/VCO_2_ slope were independent risk factor for NSCLC in predicting long‐term prognosis. The HR of age, intraoperative blood loss, VO_2_ peak, and VE/VCO_2_ slope was 2.585, 1.002, 0.988, and 1.310, respectively (in Table 3).

**TABLE 3:** Multivariate Cox regression of NSCLC.

	HR	95% CI	*p* value
Age	2.585	1.049–6.373	0.039
Intraoperative blood loss (mL)	1.002	1.000–1.004	0.024
VO^2^ peak (mL)	0.988	0.976–1.000	0.049
VE/VCO_2_ slope	1.310	1.117–1.537	0.001

### 3.5 Nomogram Construction

**FIGURE 2:**
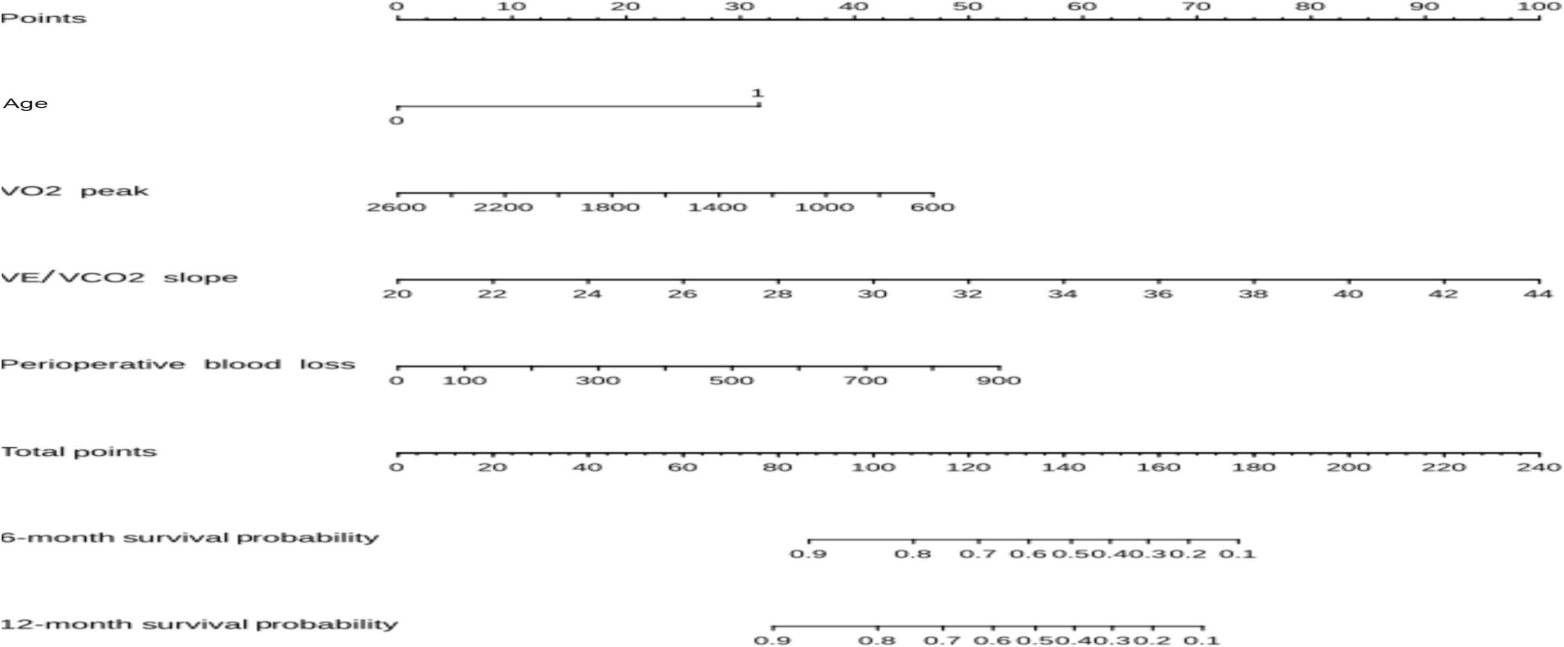
Nomogram in predicting 6‐ and 12‐month PCCs for patients with NSCLC.

The constructed model which included all independent risk factor was presented as nomogram (in Figure 2). The nomogram incorporated four factors: age, VO_2_ peak, VE/VCO_2_ slope, and intraoperative blood loss. The nomogram predicted 6‐ and 12‐month PCCs in patients with NSCLC.

### 3.6 Calibration and Internal Validation

**FIGURE 3:**
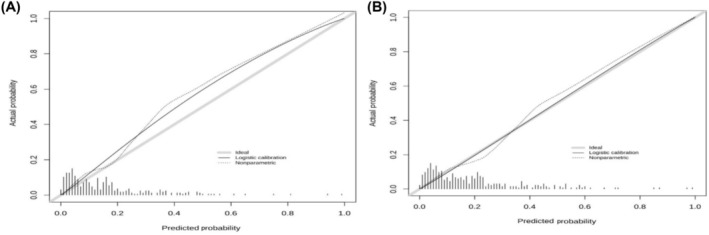
Calibration plots for predicting PCCs in 6 (A) and 12 months (B), respectively.

**FIGURE 4:**
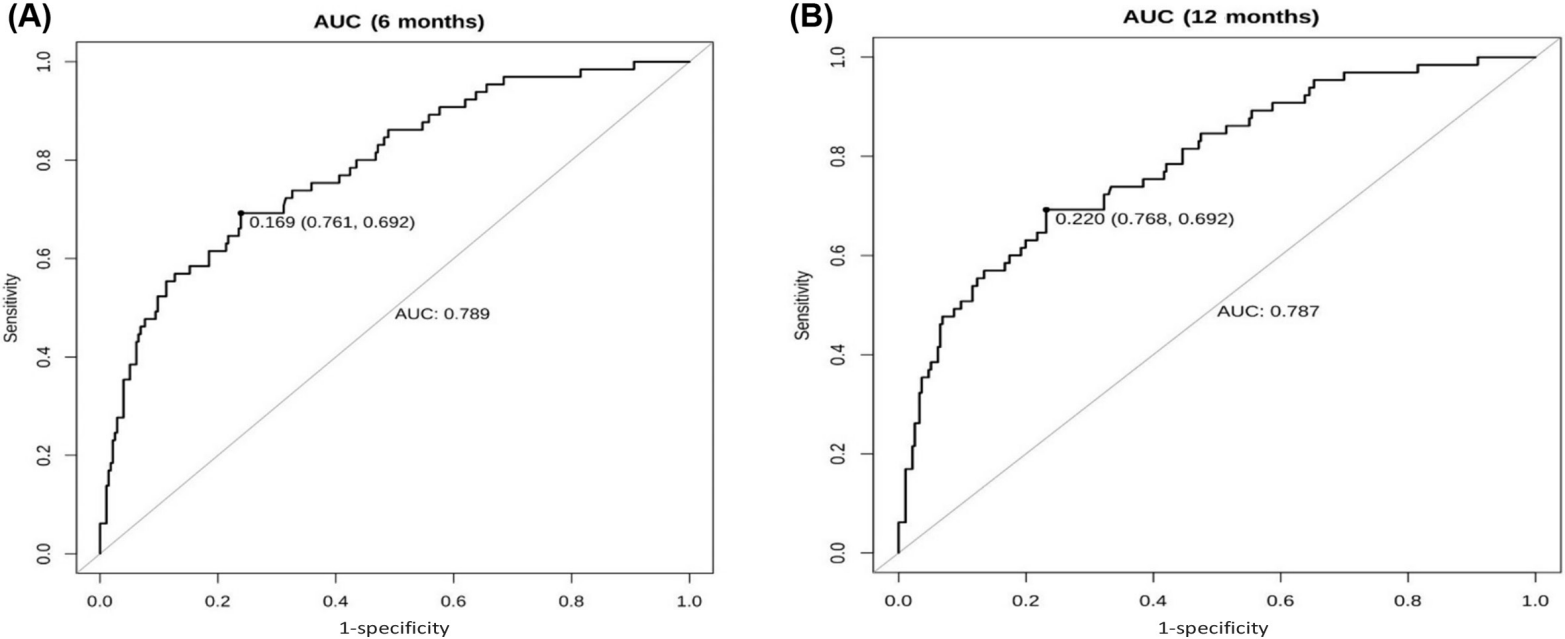
ROC curve demonstrating the discriminative ability of nomogram in 6 (A) and 12 (B) months, respectively.

**FIGURE 5:**
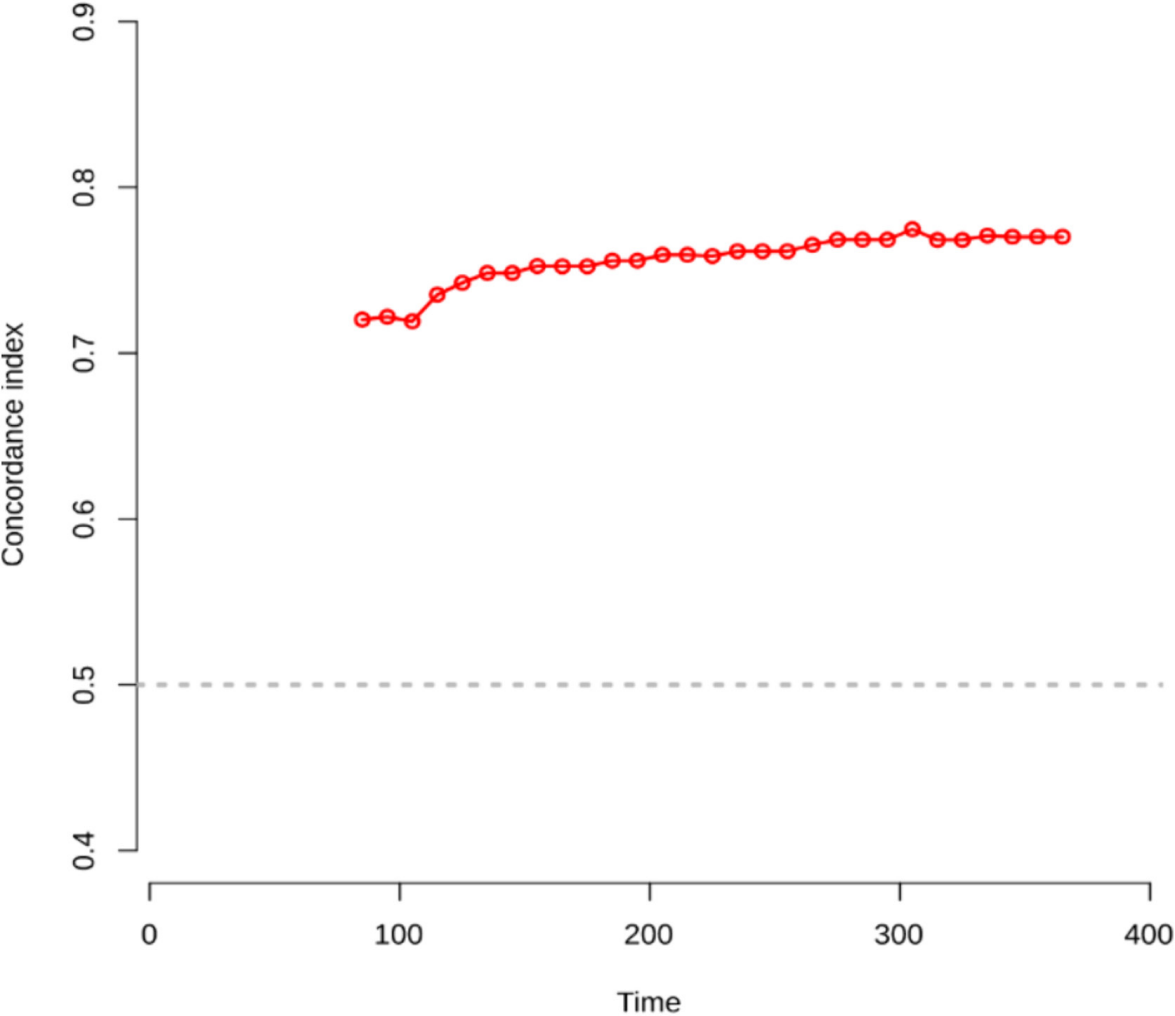
Time‐dependent ROC of AUC for nomogram in 12 months.

The calibration plot of PCCs at 6 or 12 months demonstrated good agreement between the predicted and actual probability (in Figure 3). C‐index = 0.5 is completely inconsistent, indicating that the model has no predictive effect. C‐index = 1 represents complete agreement, indicating that the predicted results of the model were completely consistent with the actual results. The C‐index of model was 0.770 (95% CI: 0.712–0.822) which showed the good discriminative and predictive value. Besides, as shown in Figure 4, the ROC curve showed that the model of nomogram has a good ability of discrimination at 6 (AUC: 0.789, 95% CI: 0.726–0.851) and 12 months (AUC: 0.787, 95% CI: 0.724–0.850) in internal validation. As seen in time‐dependent ROC (Figure 5), the discriminative ability still maintained good validity with the change of time.

## 4 Discussion

Currently, nomogram is widely used in clinical practice. Nomogram could provide more clear, easier information to understand the outcome and more accurate clinical decision [15]. Relevant clinical guidelines [7, 16] point out that the information obtained through preoperative CPET can be used to predict the postoperative morbidity and mortality, provide reference for anesthesia, and formulate exercise prescription for perioperative rehabilitation, which is conducive to improve prognosis for patients with lung cancer. Long‐term PCCs caused an adverse effect on readmission and we developed a nomogram‐incorporated CPET parameters besides clinical data in predicting long‐term prognosis to mitigate this phenomena. After analyzing the multivariate regression, we ensured four independent factors in nomogram construction, including age, intraoperative blood loss, VO_2_ peak, and VE/VCO_2_ slope.

Age is an important factor influencing prognosis for NSCLC. Langer [17] demonstrated that 60% NSCLC were more than 60 years or older. We found that older people performed poor prognosis. It is similar with previous studies [18, 19]. We speculate that poor outcome associates with comorbidity. A study of 1255 patients with NSCLC demonstrated that elder patients had more serious burden of comorbidity [20]. Comorbidity is a considerable factor affecting the poor prognosis for lung cancer, masking cancer‐related symptoms and delaying diagnosis [21]. It makes treatment more difficult and may explain poorer prognosis associated with age.

Intraoperative blood loss is an important problem which surgeons pay attention, and it reflects the invasive nature of surgery [22]. Hence, the excessive intraoperative blood loss is likely to cause poorer prognosis. Long‐term systematic hypoperfusion and impaired oxygen delivery to vital organs caused larger volumes of intraoperative blood loss [23]. The hypothalamic‐pituitary axis and autonomic nervous system are activated by the procedural stress response, which results in catabolic effects on inflammation and operative injury. Due to the large volume of intraoperative blood loss, excessive exudation caused by surgical stress response can further aggravate wild pulmonary edema and influence pulmonary artery pressure, leading to the development of PCCs [24]. Intraoperative blood loss is an independent risk factor affecting the prognosis which is in agreement with our result.

VO_2_ peak reflects the aerobic capacity of patients, which can well predict postoperative complications and mortality. It has been widely used in clinical practice. Licker et al. [25] showed that PCCs in lung cancer patients with VO_2_ peak < 10 mL/kg/min were four times higher than those with VO_2_ peak > 17 mL/kg/min. The mortality rate in lower VO_2_ peak group was 10‐fold higher compared to patients with higher VO_2_ peak [26]. VO_2_ peak can even be used as a long‐term prognostic indicator for up to 10 years for patients with lung cancer [27]. As a result, the peak level of oxygen supply capacity and exercise endurance were worse in the group with poor outcome.

VE/VCO_2_ slope has been receiving much attention in recent years. In addition, VE/VCO_2_ slope in predicting PCCs is better than VO_2_ peak [14]. Mazur et al. [28] also reached similar conclusion. They found that VE/VCO_2_ slope was better than VO_2_ peak in predicting postoperative cardiovascular complications in a study of 353 patients with lung cancer. VE/VCO_2_ slope is an indicator of ventilation efficiency, and its value is related to lung ventilation, lung perfusion, and cardiac output [29]. We consider that the increase of vascular resistance caused by the tumor itself will lead to the decrease of pulmonary ventilatory blood flow ratio, which will reduce ventilation efficiency. Most patients with lower ventilation efficiency will increase the risk and difficulty of surgery leading to bad prognosis.

There were some limitations in our study. Firstly, the study is a single‐center retrospective study, which has unavoidable selection bias. Secondly, some data such as treatment duration, methods, and genetic testing are not available due to database limitations. Besides, external validation through more databases will be required in the future. Although there are some limitations, the nomogram was the first to develop based on CPET for NSCLC in long‐term outcome.

In conclusion, we construed a nomogram with good discrimination ability in predicting PCCs. In the future, this model could apply in clinical practice to help clinicians make treatment decision and classify patients’ risk. In the meantime, external validation is needed to determine whether it could accommodate other patients.

## Author Contributions

Xinyu Wang is responsible for the conceptualization, data curation, formal analysis, investigation, methodology, supervision, validation, writing – original draft, and writing – review and editing. Jin Li is responsible for the data curation, formal analysis, investigation, conceptualization, and writing – review and editing. Min Gao, Bin Wang, Yiman Tong, and Yuhan Cao are responsible for the data curation, formal analysis, methodology, and supervision. Wei Chen is responsible for the conceptualization, project administration, and writing – review and editing.

## Conflicts of Interest

The authors declare no conflicts of interest.

## Data Availability

The data that support the findings of this study are available on request from the corresponding author (C.W.).

## References

[1] R. L. Siegel , K. D. Miller , N. S. Wagle , and A. Jemal , “Cancer Statistics, 2023,” CA: A Cancer Journal for Clinicians 73, no. 1 (2023): 17–48, 10.3322/caac.21763. 36633525

[2] W. Liang , L. Zhang , G. Jiang , et al., “Development and Validation of a Nomogram for Predicting Survival in Patients With Resected Non‐Small‐Cell Lung Cancer,” Journal of Clinical Oncology 33, no. 8 (2015): 861–869, 10.1200/JCO.2014.56.6661. 25624438

[3] J. R. Molina , P. Yang , S. D. Cassivi , S. E. Schild , and A. A. Adjei , “Non‐Small Cell Lung Cancer: Epidemiology, Risk Factors, Treatment, and Survivorship,” Mayo Clinic Proceedings 83, no. 5 (2008): 584–594, 10.4065/83.5.584. 18452692 PMC2718421

[4] M. A. Gaudet and T. A. D'Amico , “Thoracoscopic Lobectomy for Non‐Small Cell Lung Cancer,” Surgical Oncology Clinics of North America 25, no. 3 (2016): 503–513, 10.1016/j.soc.2016.02.005. 27261912

[5] X.‐E. Su , W.‐P. Hong , H.‐F. He , et al., “Recent Advances in Postoperative Pulmonary Rehabilitation of Patients With Non‐Small Cell Lung Cancer (Review),” International Journal of Oncology 61, no. 6 (2022): 156, 10.3892/ijo.2022.5446. 36321778 PMC9635865

[6] M. Tabutin , S. Couraud , B. Guibert , P. Mulsant , P.‐J. Souquet , and F. Tronc , “Completion Pneumonectomy in Patients With Cancer: Postoperative Survival and Mortality Factors,” Journal of Thoracic Oncology 7, no. 10 (2012): 1556–1562, 10.1097/JTO.0b013e31826419d2. 22982656

[7] A. Brunelli , A. W. Kim , K. I. Berger , and D. J. Addrizzo‐Harris , “Physiologic Evaluation of the Patient With Lung Cancer Being Considered for Resectional Surgery: Diagnosis and Management of Lung Cancer, 3rd ed: American College of Chest Physicians Evidence‐Based Clinical Practice Guidelines,” Chest 143, no. 5 (2013): e166S–e190S, 10.1378/chest.12-2395. 23649437

[8] M. Guazzi , R. Arena , M. Halle , M. F. Piepoli , J. Myers , and C. J. Lavie , “2016 Focused Update: Clinical Recommendations for Cardiopulmonary Exercise Testing Data Assessment in Specific Patient Populations,” European Heart Journal 39, no. 14 (2018): 1144–1161, 10.1093/eurheartj/ehw180. 27141094

[9] T. Chen , X. Zhan , J. Du , et al., “A Simple‐To‐Use Nomogram for Predicting Early Death in Metastatic Renal Cell Carcinoma: A Population‐Based Study,” Frontiers in Surgery 9 (2022): 871577, 10.3389/fsurg.2022.871577. 35392061 PMC8980350

[10] B. Jia , Q. Zheng , J. Wang , et al., “A Nomogram Model to Predict Death Rate Among Non‐Small Cell Lung Cancer (NSCLC) Patients With Surgery in Surveillance, Epidemiology, and End Results (SEER) Database,” BMC Cancer 20, no. 1 (2020): 666, 10.1186/s12885-020-07147-y. 32680464 PMC7367407

[11] Z. Zuo , G. Zhang , P. Song , et al., “Survival Nomogram for Stage IB Non‐Small‐Cell Lung Cancer Patients, Based on the SEER Database and an External Validation Cohort,” Annals of Surgical Oncology 28, no. 7 (2021): 3941–3950, 10.1245/s10434-020-09362-0. 33249521

[12] ATS/ACCP Statement on cardiopulmonary exercise testing , “ATS/ACCP Statement on Cardiopulmonary Exercise Testing,” American Journal of Respiratory and Critical Care Medicine 167, no. 2 (2003): 211–277. 12524257 10.1164/rccm.167.2.211

[13] I. Bouabdallah , V. Pauly , M. Viprey , et al., “Unplanned Readmission and Survival After Video‐Assisted Thoracic Surgery and Open Thoracotomy in Patients With Non‐Small‐Cell Lung Cancer: A 12‐Month Nationwide Cohort Study,” European Journal of Cardio‐Thoracic Surgery 59, no. 5 (2021): 987–995, 10.1093/ejcts/ezaa421. 33236091

[14] H. Shafiek , J. L. Valera , B. Togores , J. A. Torrecilla , J. Sauleda , and B. G. Cosio , “Risk of Postoperative Complications in Chronic Obstructive Lung Diseases Patients Considered Fit for Lung Cancer Surgery: Beyond Oxygen Consumption,” European Journal of Cardio‐Thoracic Surgery 50, no. 4 (2016): 772–779, 10.1093/ejcts/ezw104. 27059429

[15] L. Wang , L. Wu , J. Liu , et al., “Prognostic Nomogram for Surgery of Lung Cancer in HIV‐Infected Patients,” Journal of Thoracic Disease 13, no. 1 (2021): 76–81, 10.21037/jtd-20-2268. 33569187 PMC7867813

[16] D. Z. H. Levett , S. Jack , M. Swart , et al., “Perioperative Cardiopulmonary Exercise Testing (CPET): Consensus Clinical Guidelines on Indications, Organization, Conduct, and Physiological Interpretation,” British Journal of Anaesthesia 120, no. 3 (2018): 484–500, 10.1016/j.bja.2017.10.020. 29452805

[17] C. J. Langer , “Elderly Patients With Lung Cancer: Biases and Evidence,” Current Treatment Options in Oncology 3, no. 1 (2002): 85–102. 12057091 10.1007/s11864-002-0045-9

[18] Y. J. Cho , Y. M. Cho , S. H. Kim , K.‐H. Shin , S.‐T. Jung , and H. S. Kim , “Clinical Analysis of Patients With Skeletal Metastasis of Lung Cancer,” BMC Cancer 19, no. 1 (2019): 303, 10.1186/s12885-019-5534-3. 30943924 PMC6446278

[19] M. Agarwal , G. Brahmanday , G. W. Chmielewski , R. J. Welsh , and K. P. Ravikrishnan , “Age, Tumor Size, Type of Surgery, and Gender Predict Survival in Early Stage (Stage I and II) Non‐Small Cell Lung Cancer After Surgical Resection,” Lung Cancer 68, no. 3 (2010): 398–402, 10.1016/j.lungcan.2009.08.008. 19762109

[20] T. R. Asmis , K. Ding , L. Seymour , et al., “Age and Comorbidity as Independent Prognostic Factors in the Treatment of Non Small‐Cell Lung Cancer: A Review of National Cancer Institute of Canada Clinical Trials Group Trials,” Journal of Clinical Oncology 26, no. 1 (2008): 54–59, 10.1200/JCO.2007.12.8322. 18165640

[21] M. Bjerager , T. Palshof , R. Dahl , P. Vedsted , and F. Olesen , “Delay in Diagnosis of Lung Cancer in General Practice,” British Journal of General Practice: The Journal of the Royal College of General Practitioners 56, no. 532 (2006): 863–868. 17132354 PMC1927095

[22] H. Nakamura , H. Saji , N. Kurimoto , T. Shinmyo , and R. Tagaya , “Impact of Intraoperative Blood Loss on Long‐Term Survival After Lung Cancer Resection,” Annals of Thoracic and Cardiovascular Surgery 21, no. 1 (2015): 18–23, 10.5761/atcs.oa.13-00312. 24583702 PMC4989982

[23] J. I. Cué , J. C. Peyton , and M. A. Malangoni , “Does Blood Transfusion or Hemorrhagic Shock Induce Immunosuppression?” Journal of Trauma 32, no. 5 (1992): 613–617. 1588650 10.1097/00005373-199205000-00013

[24] S. Li , K. Zhou , Y. Lai , C. Shen , Y. Wu , and G. Che , “Estimated Intraoperative Blood Loss Correlates With Postoperative Cardiopulmonary Complications and Length of Stay in Patients Undergoing Video‐Assisted Thoracoscopic Lung Cancer Lobectomy: A Retrospective Cohort Study,” BMC Surgery 18, no. 1 (2018): 29, 10.1186/s12893-018-0360-0. 29792183 PMC5966911

[25] M. Licker , J. M. Schnyder , J. G. Frey , et al., “Impact of Aerobic Exercise Capacity and Procedure‐Related Factors in Lung Cancer Surgery,” European Respiratory Journal 37, no. 5 (2011): 1189–1198, 10.1183/09031936.00069910. 20847073

[26] A. Brunelli , R. Belardinelli , M. Refai , et al., “Peak Oxygen Consumption During Cardiopulmonary Exercise Test Improves Risk Stratification in Candidates to Major Lung Resection,” Chest 135, no. 5 (2009): 1260–1267, 10.1378/chest.08-2059. 19029436

[27] J. Lindenmann , N. Fink‐Neuboeck , M. Fediuk , et al., “Preoperative Peak Oxygen Consumption: A Predictor of Survival in Resected Lung Cancer,” Cancers (Basel) 12, no. 4 (2020): 836, 10.3390/cancers12040836. 32244329 PMC7226454

[28] A. Mazur , K. Brat , P. Homolka , et al., “Ventilatory Efficiency Is Superior to Peak Oxygen Uptake for Prediction of Lung Resection Cardiovascular Complications,” PLoS ONE 17, no. 8 (2022): e0272984, 10.1371/journal.pone.0272984. 35960723 PMC9374210

[29] P. L. Parazzi , F. A. Marson , M. A. Ribeiro , C. I. Schivinski , and J. D. Ribeiro , “Ventilatory Efficiency in Children and Adolescents: A Systematic Review,” Disease Markers 2015 (2015): 546891, 10.1155/2015/546891. 26063959 PMC4434182

